# Clinical Characteristics of COVID-19-Related Reversible Cerebral Vasoconstriction Syndrome: A Systematic Review of Case Series

**DOI:** 10.3390/jcm14020487

**Published:** 2025-01-14

**Authors:** Raon Jung, Yun-Seo Oh, Soyoun Choi, Moo-seok Park, Hee-Jung Ha, Na Young Kim, Sohee Wang, Hosseini Seyedehmahla, Yoonkyung Chang, Tae-Jin Song

**Affiliations:** 1 Ewha Womans University College of Medicine, Seoul 03760, Republic of Korea; laonkingg@gmail.com (R.J.); yoonsoh20@gmail.com (Y.-S.O.); omg24202@ewha.ac.kr (S.W.); mahlalee94@ewha.ac.kr (H.S.); 2 Department of Neurology, Seoul Hospital, Ewha Womans University College of Medicine, Seoul 07804, Republic of Korea; applethdus@gmail.com (S.C.); pierceu@hanmail.net (M.-s.P.); milly0402@naver.com (H.-J.H.); 3 Department of Neurology, Mokdong Hospital, Ewha Womans University College of Medicine, Seoul 07985, Republic of Korea; kimnyny122585@gmail.com

**Keywords:** reversible cerebral vasoconstriction syndrome, COVID-19 infection, stroke, prognosis

## Abstract

**Background and Objectives:** Although reversible cerebral vasoconstriction syndrome (RCVS) is a rare disease, the condition may occur with COVID-19 infection. We aimed to investigate the clinical characteristics of RCVS through a systematic review of case reports and case series that reported on COVID-19-related RCVS. **Methods**: A literature search was performed in PubMed (MEDLINE), SCOPUS, and Web of Science. The search was confined to articles published between 17 November 2019 and 14 August 2024. The search terms were (“COVID-19” OR “SARS CoV-2”) AND (“RCVS” OR “Reversible cerebral vasoconstriction syndrome”). The search protocol was registered in PROSPERO (CRD42024491818). A total of twenty-four cases were included, nine case series consisting of nineteen cases and five cases from our hospital. Clinical characteristics were investigated, including risk factors, symptoms, brain and angiographic findings, treatment options, and prognoses. **Results:** The average age was 37.1 years, and females comprised 70.8% of the cohort. COVID-19 vaccination was administered in five cases. Vasoconstriction was most frequently noted in middle cerebral arteries (90.9%). Among the included patients, 12 (50.0%) experienced strokes as a complication of RCVS, and the mortality rate was 9.1%. Follow-up imaging findings were available for 14 of the 24 cases (58.3%). Among these, vasoconstriction was fully improved in 64.3%, partially improved in 28.6%, and aggravated in 7.1%. **Conclusions:** While the recovery rate of vasoconstriction on brain angiographic findings was not uncommon, our systematic review indicates a potential for a relatively poor neurological prognosis in COVID-19-related RCVS.

## 1. Introduction

SARS-CoV-2 (COVID-19) is a highly contagious respiratory disease that originated from Wuhan, China in December 2019 and has spread worldwide. As of 14 August 2024, there were 775.83 million confirmed COVID-19 cases and 7.06 million COVID-19-related deaths. Common symptoms of COVID-19 infection include fever, cough, fatigue, dyspnea, and sputum production [1]. COVID-19 is now endemic, and various systemic complications, including neurological complications, caused by COVID-19 continue to be a global issue [2,3].

Reversible cerebral vasoconstriction syndrome (RCVS) is a condition marked by the temporary constriction of cerebral arteries, typically resolving within three months. RCVS is often associated with severe headaches, including thunderclap headaches, and various neurological complications like ischemic stroke, hemorrhagic stroke, subarachnoid hemorrhage (SAH), seizure, and posterior reversible encephalopathy syndrome (PRES) [4,5,6,7,8,9]. The pathogenesis of RCVS is not well understood, but the transient failure of regulation of cerebral arterial tone with sympathetic overactivity may play an important role [8]. Although RCVS can occur spontaneously, most cases are associated with precipitating factors such as the use of vasoactive agents, cough, sexual intercourse, vigorous exercise, a history of migraine, systemic lupus erythematosus, carotid dissection, and/or pregnancy [9,10].

COVID-19 infection can cause an excessive immune response that may induce various systemic complications, including venous thromboembolism, myocarditis, arrhythmia, myalgia, and dermatitis [11]. Given the proposal that the destruction of the blood–brain barrier due to inflammation is a plausible cause of RCVS [12], COVID-19 infection could be a potential trigger for RCVS. However, RCVS-associated COVID-19 cases have rarely been reported [13,14,15,16]. Therefore, we aimed to conduct a systematic review of case reports to investigate the clinical characteristics of RCVS associated with COVID-19 infection.

## 2. Materials and Methods

### 2.1. Protocol

We followed the Preferred Reporting Items for Systematic Reviews and Meta-Analyses (PRISMA) reporting guidelines to extract data and assess the validity of this systematic review of the case series. The IRB (IRB number: EUMC 2023-10-012) waived the requirement for informed consent due to the retrospective nature of this study and the use of anonymized patient data. We ensured that all data were de-identified to maintain patient confidentiality and objectivity. Furthermore, we registered the systematic review protocol for cases that were published in PROSPERO (CRD42024491818).

### 2.2. Search Strategy and Eligibility Criteria

We searched PubMed (MEDLINE), SCOPUS, and Web of Science. The search was confined to articles published between 17 November 2019, the date of the first reported COVID-19 infection in Wuhan, and 14 August 2024. We restricted the search to human and case studies written in English. The search terms were (“COVID-19” OR “SARS CoV-2”) AND (“RCVS” OR “Reversible cerebral vasoconstriction syndrome”) (Supplementary Table S1).

The inclusion criteria of our study were articles on RCVS occurrence after COVID-19 infection and articles with case series, case reports, and systematic or narrative reviews. Only narrative reviews that included case series or reports were used.

The exclusion criteria were articles on cases without histories of COVID-19 infection, articles that did not cover RCVS, letters or commentaries that did not include case series or case reports, articles for which the full text was inaccessible, cases of only vaccination without infection, and cases without sufficient evidence of RCVS.

### 2.3. Study Selection and Data Extraction

**Figure 1:**
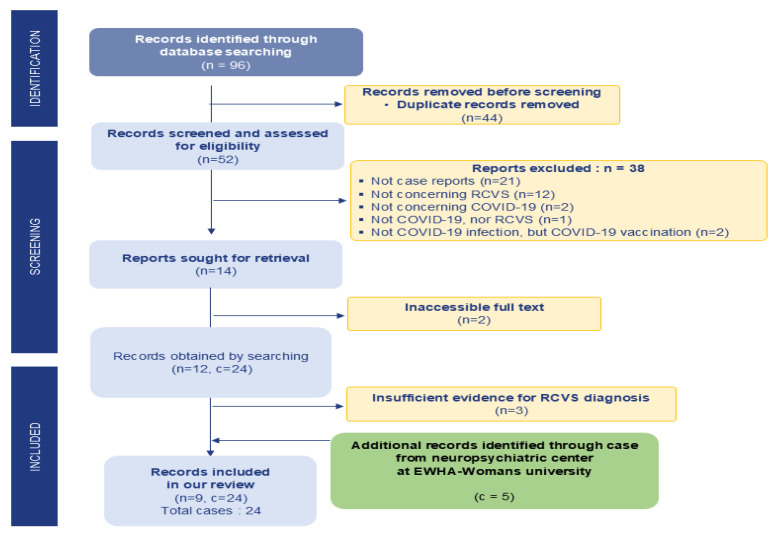
The strategy for selecting articles. There were 19 cases extracted from 9 articles. We added 5 of our hospital’s cases to this and investigated a total of 24 cases. n = number of articles; c = cases.

Two investigators (Y-S.O. and R-O.J.) independently screened the articles, and discrepancies were resolved by discussion with a third investigator (T-J.S.). Duplications were eliminated, and initial screening involved reviewing titles and abstracts to exclude articles that did not meet the selection criteria. Subsequently, inaccessible articles were removed. Additional cases were identified from the neuropsychiatric center at Ewha Womans University. We selected cases diagnosed with RCVS by neurologists following COVID-19 infection from 1 January 2020 to 31 December 2023. The strategy for selecting articles is shown in Figure 1.

Patient data were extracted by three authors (Y-S.O., R-O.J. and Y-C). The extracted data were entered into a database. These data included age, sex, ethnicity, relevant medical history, relevant medication history, COVID-19 vaccination status, initial blood pressure, risk factors associated with RCVS, confirmatory test for COVID-19 diagnosis, COVID-19-related symptoms, RCVS-related symptoms, and complications of RCVS. Also extracted were RCVS2 score data and laboratory and other test results. These tests included blood tests, cerebrospinal fluid analyses, electrocardiograms, and electroencephalograms. The severity of COVID-19, as determined by the need for admission to the intensive care unit for COVID-19-related respiratory complications and treatments for RCVS or other medical conditions, was assessed. Radiologic findings during hospitalization included CT, MRI, CTA, MRA, and digital subtraction angiography (DSA), and results were extracted. The outcomes of discharge including modified Rankin scale (mRS), follow-up period, brain imaging follow-up findings, and prognoses were also investigated.

Among the significant associated or risk factors of RCVS, the characteristics of previously diagnosed primary headache, hypertension, smoking history, pregnancy, use of a vasoactive agent, sexual intercourse, and previous vascular or autoimmune disease, such as Takayasu’s arteritis or systemic lupus erythematosus, were included [17,18,19]. A history of multisystem inflammatory syndrome in children and Kawasaki syndrome was also considered significant. Possible offending drugs for the occurrence of RCVS are listed in Supplementary File S1.

The complications of RCVS were confined to cerebral lesions that could be identified through brain imaging; these lesions included PRES, cerebral edema, ischemic stroke, cerebral infarction, and intracerebral hemorrhage [13].

RCVS2 score is a tool for diagnosing or excluding RCVS from other intracranial arteriopathies at admission. Five points were assigned for the presence of a recurrent or single thunderclap headache; two points were given if the carotid artery was involved; three points were given for the presence of a vasoconstrictive trigger; one was given a point if the patient was a woman, and one point was assigned for the presence of subarachnoid hemorrhage. These scores were added, and a total score of ≥5 indicated 90% sensitivity and a 99% specificity for diagnosing RCVS. A score ≤ 2 indicated 85% sensitivity and a 100% specificity for excluding RCVS. Scores of 3 and 4 indicated 10% sensitivity and an 86% specificity for diagnosing RCVS [20]. In our cases, the RCVS2 score was calculated from the given information when not specifically presented. While the score was validated for ages from 18 to 55 in the study [20], we extended the age range and applied it to all cases in our study.

The involvement of cerebral arteries was identified through CTA, MRA, and DSA findings. The blood vessels assessed included the internal carotid artery, vertebral artery, vertebrobasilar junction, basilar artery, superior cerebellar artery, anterior inferior cerebellar artery (AICA), posterior inferior cerebellar artery (PICA), anterior cerebral artery (ACA), middle cerebral artery (MCA), and posterior cerebral artery (PCA). If vessel images were presented instead of specific statements, we estimated the involved segments. When only the proximal or distal portions of the ACA, MCA, and PCA were mentioned without specific segments, we classified proximal as A1, M1, and P1 and distal as A3, M3, and P3 or more distal.

Treatments included the administration of a vasodilator, analgesics, steroid, and surgery. The treatments were divided into RCVS treatment and treatment for other medical conditions.

Prognosis was divided into clinical prognosis and imaging prognosis. Clinical prognosis was assessed using an mRS. In cases where an mRS was not detailed, the neurological status described in this article was used. Imaging prognosis was evaluated by investigating the follow-up imaging findings.

### 2.4. Quality Assessment

Since our study only collected case reports, a detailed quality assessment could not be performed.

### 2.5. Data Analysis

This study included the collection of case reports, primarily using descriptive statistical analysis. Additionally, Chi-square or Fisher’s exact test was used to analyze the statistical significance of the categorical variables, with a *p*-value less than 0.05 considered statistically significant. All statistical analyses were conducted using the R software (version 4.3.1).

## 3. Results

A total of 12 articles with 22 cases were identified. Three cases ([21,22,23]) were additionally excluded (Supplementary Table S2). One case showed a persisting vasospasm in repeated angiogram and eventually died, which is different from the typical course of RCVS [21]. Another was excluded because an angiogram result was not present [22]. The final case was excluded because there was no evidence of vascular malformation in the angiogram [23]. Therefore, a comprehensive analysis was conducted on 19 cases. The five additional cases were those admitted to the Department of Neurology at Ewha Womans University of Seoul Hospital.

### 3.1. Demographic Data of Patients Group

The ages of the patients ranged from 6 to 62 years, with a median of 37 years (30.75–47.25 years) and an average of 37.0 years (±14.2 years). Since patient #2’s age was described as “in her thirties”, she was assumed to be 35 years old. Seventeen patients (70.8%) were female. Among the 16 patients with race information, six were Caucasian, five were Asian, four were African American, and one was Haitian.

Blood pressure was mentioned in 20 cases, and specific blood pressure values were provided in 18 cases. Of the remaining two patients, one was described as having elevated blood pressure during a physical examination. The other was described as having blood pressure within the normal range. In most cases, the initial blood pressure was measured in the emergency room or at the time of hospitalization. The mean systolic blood pressure was 162.2 mmHg (±42.7); the mean diastolic blood pressure was 87.0 mmHg (±21.2), and the mean arterial pressure was 108.8 mmHg (±24.4). One diastolic blood pressure value was missing.

### 3.2. Risk Factors of RCVS

#### 3.2.1. Female

Among our patient group, 17 of the 24 patients (70.8%) were female.

#### 3.2.2. Use of Vasoactive Agents

In our patient group, 20 patients had a history of taking prescribed medications. Among these, 17 patients (85.0%) were medicated with drugs that could be potential risk factors for RCVS (Supplementary Table S3).

#### 3.2.3. History of Headache Disorders

In our patient group, nine patients (37.5%) had a history of headaches or migraines. Among these, eight (33.3%) had a history of migraines, and one (4.2%) had a history of “stabbing” headache.

#### 3.2.4. Vaccination Status

COVID-19 vaccination status was described in six cases, of which five were vaccinated. All of them were vaccinated with BNT162b2 (Pfizer, New York, NY, USA).

#### 3.2.5. Other Associated Conditions

Six patients (25.0%) had a history of hypertension. Of these, two (8.3%) were current smokers; two (8.3%) suffered from vascular disease, one with Kawasaki shock syndrome and the other with multisystem inflammatory syndrome in children. Additional information regarding the demographic data is presented in Table 1.

### 3.3. Clinical Features

#### 3.3.1. COVID-Related Symptoms

Among the 24 cases, common COVID-19-related symptoms included fever (nine cases, 37.5%), cough (seven cases, 29.2%), dyspnea (five cases, 20.8%), sore throat (five cases, 20.8%), and general weakness (five cases, 20.8%). The other symptoms are presented in Supplementary Table S4. Six of the twenty-four cases (25.0%) required admission to an intensive care unit due to the severity of their COVID-19 condition during hospitalization.

#### 3.3.2. RCVS-Related Symptoms

Common RCVS-related symptoms included twenty cases of thunderclap headache and non-specific headache (83.3%), nine cases of nausea (37.5%), eight cases of vomiting (33.3%), three cases of visual impairment (12.5%), three cases of photophobia (12.5%), three cases of confusion (12.5%), three cases of encephalopathy (12.5%), and three cases of hemiplegia (12.5%). The other symptoms are presented in Supplementary Table S5. The other clinical features of COVID-19-related RCVS are demonstrated in Table 2.

#### 3.3.3. Temporal Relationship Between COVID-19 Symptoms and RCVS Diagnosis

COVID-19 symptoms preceded RCVS diagnosis in 20 of the 24 cases. The remaining four cases were asymptomatic for COVID-19 but were diagnosed with both RCVS and COVID-19 infection.

### 3.4. Diagnosis

#### 3.4.1. Confirmatory Test for COVID-19 Diagnosis

COVID-19 infection was confirmed in 23 of the 24 cases (95.8%). Among these 23 confirmed cases, 19 were identified through polymerase chain reaction (PCR). Among the remaining four confirmed cases, three were identified as positive without any record of a specific testing method. One case tested positive for IgG antibodies in serological tests. The unconfirmed case had recent contact with a COVID-19 patient a week prior to hospitalization.

#### 3.4.2. RCVS2 Score

The mean RCVS2 score was 6.3 (±3.3); the median score was 6.5 (5.75–9). If confined to patients aged 18–55 years, the mean was 6.2 (±3.5), and the median was 6 (5–9). We considered three cases as RCVS-positive despite having RCVS2 scores < 2, which included patients #9, #12, and #18. Patient #9′s DSA findings included diffuse vasculopathy of the M2 and M3 division. Patient #12′s CTA findings included diffuse intracranial vascular abnormalities, vasospasm, or vasculitis; patient #18′s CTA and DSA findings included the narrowing of the terminal parts of both the ICA and bilateral MCAs with pruning of the distal flow.

### 3.5. Radiologic Findings

#### 3.5.1. Brain CT and MRI Findings

Brain CT/MRI findings were presented in 19 of the 24 cases (79.2%). The main abnormalities on brain CT included subarachnoid hemorrhage (SAH), parenchymal hemorrhage, and vasogenic edema. The typical abnormal findings on MRI included SAH, subdural hemorrhage (SDH), cerebral infarction, edema, gyral swelling, and sulcal effacement. Hemorrhage was found in eight of nineteen cases (42.1%); ischemic lesions were found in six (31.6%).

#### 3.5.2. Brain CTA, MRA, DSA

Among the twenty-four cases, one case showed unremarkable abnormalities in cerebral blood vessels. Additionally, in case 12, the involved cerebral vascular segment could not be determined. Therefore, the analysis of brain angiographic findings was conducted with 22 cases.

The frequencies of involved cerebral vessels were as follows: MCA, twenty cases, 90.9%; ACA, fifteen cases, 68.2%; PCA, thirteen cases, 59.1%; internal carotid artery, four cases, 18.2%; basilar artery, three cases, 13.6%; AICA, two cases, 9.1%; PICA, two cases, 9.1%; vertebral artery, one cases, 4.5%; vertebrobasilar junction, one case, 4.5%; and superior cerebellar artery, one case, 4.5%. These findings were confirmed through CTA, MRA, and DSA (Supplementary Table S6). Only the anterior cerebral artery showed more involvement on the right side; The vertebral artery, anterior inferior cerebellar artery, and superior cerebellar artery, were equally involved on both sides; the remaining cerebral vessels showed more involvement on the left side.

Among the fifteen cases involving the ACA, the A1, A2, and A3 segments were involved in three (13.6%), ten (45.5%), and one (4.5%) case, respectively. There were three cases in which the involved segments were not specified or mentioned. Notably, the A2 segment was predominantly affected. On the right ACA, the A1, A2, and A3 segments were invaded in three (13.6%), ten (45.5%), and zero cases, respectively. The left ACA showed invasion in three (13.6%), eight (36.4%), and one (4.5%) case for A1, A2, and A3 segments, respectively.

Among the twenty cases involving the MCA, the M1, M2, M3, and M4 segments were involved in six (27.3%), twelve (54.5%), six (27.3%), and five (22.7%) cases, respectively. There were two cases in which the involved segments were not specified or mentioned. Notably, the M2 segment was predominantly affected. On the right MCA, the M1, M2, M3, and M4 segments were involved in four (18.2%), ten (45.5%), three (13.6%), and two (9.1%) case, respectively. Similarly, on the left MCA, involvement occurred in five (22.7%), twelve (54.5%), six (27.3%), and five (22.7%) cases, respectively, for M1, M2, M3, and M4 segments.

**Figure 2:**
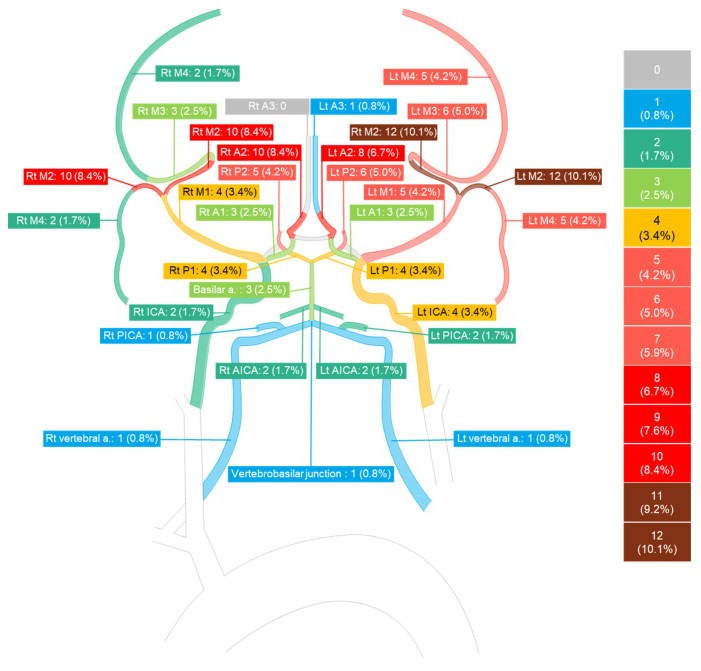
Frequency of involved cerebral vascular segments. Rt = right and Lt = left. Color coding according to frequency is noted in the figure. The number (%) written next to each segment indicates the frequency and the ratio (Number_case_/Number_total number of involved segments_). The total number of cerebral vessels involvement sites in our patients as determined by radiology was 119.

Among the thirteen cases involving the PCA, the P1, P2, and P3 segments were involved in four (18.2%), seven (31.8%), and four (18.2%) cases, respectively. In three cases, the involved segment was not specified. Notably, the P2 segment was predominantly affected. On the right PCA, involvement was observed in four (18.2%), six (27.3%), and three (13.6%) cases for P1, P2, and P3 segments, respectively. The left PCA exhibited invasion in four (18.2%), six (27.3%), and four (18.2%) cases for P1, P2, and P3 segments, respectively (Figure 2).

#### 3.5.3. Complications

Of the 24 patients in our study, 13 (54.2%) had complications from RCVS and 12 (50.0%) had stroke. Intracerebral hemorrhage (SAH, parenchymal hemorrhage, and subdural hemorrhage) was found in eight cases; cerebral infarction was found in six cases; PRES was found in two cases, and cerebral edema was found in two cases.

### 3.6. Treatment

Among the 24 patients, 13 patients (54.2%) received vasodilators as treatment. Specifically, nimodipine was prescribed to nine patients, amlodipine to five patients, nicardipine to four patients, verapamil to three patients, nifedipine to one patient, milrinone to one patient, and fimasartan to five patients. Aspirin was prescribed to three patients (12.5%) as an anti-thrombotic. Steroids were prescribed to eight patients (33.3%). Dexamethasone was prescribed to four patients, methylprednisolone to three patients, and prednisone to one patient. Anticonvulsant (levetiracetam) was prescribed to one patient (4.2%). As for surgical treatments, two decompressive craniectomies were performed. The other treatments are listed in Table 3.

### 3.7. Clinical Prognosis

Table 3 includes clinical prognosis for the included cases. Of the 24 cases, an mRS was suggested in only 12 cases. Among the 12 cases in which an mRS was not described, we could assume an mRS in 10 cases based on the neurological condition described in this article. The mRS of 19 patients was measured at discharge. One had the assessment at one month after discharge, one patient at two months after discharge, and one patient at three months after discharge. Among the twenty-two patients with an available mRS, fourteen patients (63.6%) had an mRS of zero; two patients (9.1%) had an mRS of one; two patients (9.1%) had an mRS of two; one patient (4.5%) had an mRS of three; one patient (4.5%) had an mRS of five; and two patients (9.1%) had an mRS of six.

Of the twenty-four patients, twenty (83.3%) were discharged, two (8.3%) died, and two (8.3%) were hospitalized at the time of article publication. Patients #1, #14, and #18 were not explicitly designated as discharged in the records. Symptoms were resolved for patients #1 and #14, and patient #18 showed significant improvement. Therefore, these cases were classified as discharged. Among the twenty discharged patients, six (25.0%) had neurological disorders or sequelae, thirteen (54.2%) recovered without sequelae, and the clinical prognosis was not indicated in one case. The number of cases with specific neurological sequalae was two of six (#17 and #21). Patients #21 had a visual impairment such as visual field defect. Mild right-sided ataxia and dysarthria remained in patient #17.

To further investigate potential associations between patient characteristics and disease severity, we performed logistic regression analyses with ICU admission as the dependent variable defining severity. Independent variables included age, sex, the presence of complications, a history of hypertension, and the use of offending drugs associated with RCVS. However, the analyses did not yield any statistically significant findings (Table 4). Supplementary Tables S7 and S8 present the odds ratio analyses for mortality and complications in RCVS. Notably, male sex was significantly associated with complications, with an odds ratio of 12.670 (95% CI [1.119, 712.230], *p* = 0.023).

### 3.8. Imaging Prognosis

Follow-up and the prognosis data of the images are described in Table 3. In 14 of the 24 cases (58.3%), the recovery or aggravation of vascular abnormalities could be investigated through radiologic findings. The outcomes of these 14 cases were categorized into three groups: aggravated, partially improved, and fully improved. nine patients of fourteen (64.3%) fully improved, four patients partially improved (28.6%), and one patient’s (#8) outcome aggravated. Patient #8 showed residual subarachnoid hemorrhage and a new onset of small subdural hemorrhage on the follow-up image.

## 4. Discussion

In this study, through a systematic review, we investigated the published case reports or case series of COVID-19 infection-related RCVS. Consequently, we identified the clinical characteristics and prognosis of COVID-19 infection-related RCVS.

### 4.1. Demographic Data of the Patient Group

RCVS usually occurs in the extensive range of ages from 4 months to 80 years old, and the incidence tends to peak at 42 years old [4,29,30,31]. Additionally, 70.7–89.6% of RCVS patients are female [17]. In our study, the mean age of our patient group was about four years younger compared to the COVID-19-unrelated RCVS patients [20]. The sex distribution for RCVS was similar between the patients in our study and the COVID-19-unrelated RCVS patients.

### 4.2. Risk Factors

About 79.1–88.5% of patients with COVID-19-unrelated RCVS have precipitating factors [17,31], specifically involving vasoactive drug use (41.4%), being pregnant or post-partum (20.9%), and engaging in sexual intercourse (10.5%) [17]. In our review, we had a relatively high frequency of individuals using offending drugs and a low frequency of pregnancy and engaging in sexual intercourse compared to previous reports. These results suggest that COVID-19-unrelated and COVID-19-related RCVS may have different triggering factors. However, since our study included a small sample size and only case reports, caution should be taken in the interpretation of these results.

### 4.3. Discussion on Clinical Features

In previous studies, symptoms related to COVID-19 infection varied [1,6,32]. In our study, sore throat, headache, and diarrhea were more frequently noted than in previous studies. However, other COVID-19-related symptoms were relatively less frequently noted compared to previous studies.

Symptoms related to RCVS included thunderclap headache and non-specific headache (93.2%), motor weakness (36.6%), visual disturbances (30.4%), altered consciousness (20.4%), seizures (17.3%), nausea (26.7%), and vomiting (16.8%) [17]. In our study, headache and visual disturbance were the most common symptoms related to RCVS, and these results were in line with previous studies. Nausea and vomiting were more common in our data set compared to the COVID-19-unrelated RCVS cases. This discrepancy may be caused by differences in the characteristics of the included patients or by the COVID-19 infection’s symptoms. There was one case (#8) in which the occurrence of COVID-19 symptoms did not precede RCVS diagnosis. The patient developed COVID-19 symptoms within 48 h after RCVS diagnosis. Considering that the average incubation period for COVID-19 is about six days [33,34], COVID-19 infection would have preceded RCVS diagnosis in this patient.

In our study, four patients were asymptomatic for COVID-19 and developed RCVS. This finding suggests that RCVS can occur even in the absence of typical COVID-19 symptoms. The occurrence of RCVS in asymptomatic COVID-19 patients highlights the possibility that subclinical infection or virus-induced endothelial dysfunction may trigger cerebral vasoconstriction.

The follow-up data varied among the cases, with some patients having extended follow-up periods of up to eight months, while others lacked detailed long-term outcome information (Table 3). This variability poses challenges in comprehensively evaluating the long-term prognosis of RCVS in the context of COVID-19.

### 4.4. Complications

Cerebral ischemic lesions (47.6%) and cerebral hemorrhage (35.1%) are the leading parenchymal abnormalities in RCVS [17]. In our study, cerebral hemorrhage was more common than cerebral ischemic lesions. This may be due to increased coagulopathy or bleeding tendency, the rupture of blood vessels, the disruption of the blood–brain barrier, or uncontrolled hypertension due to COVID-19 [35,36,37,38].

### 4.5. Angiographic Findings

In our study, the MCA was the most frequently involved vessel, followed by the ACA and PCA. The involved cerebral vascular segments and their frequencies of involvement were similar to those previously reported [20]. Therefore, the invaded vessels in COVID-19-related RCVS and COVID-19-unrelated RCVS are not significantly different.

Generally, in RCVS, there is a mismatch between neurological symptoms such as headache and cerebral angiographic findings; multivessel stenosis and vasospasm in angiographic findings may be delayed compared to neurological symptoms such as headache [8]. In our cases, the timing of cerebral angiographic findings was not homogeneous, possibly due to the characteristics of RCVS-related COVID-19 infection. Comparing the angiographic course of COVID-19-unrelated RCVS to COVID-19-related RCVS over time is therefore difficult. However, considering that the mean number of involved cerebral vascular segments was similar to that of a previous study [29], the pattern and course of cerebrovascular invasion between COVID-19-unrelated RCVS and COVID-19-related RCVS may not be significantly different.

### 4.6. Clinical Prognosis

Generally, the prognosis of RCVS is favorable. Headaches and radiologic abnormal findings are resolved within three months. In a previous study among COVID-19-unrelated RCVS patients, when short-term prognosis was evaluated using the mRS, clinical prognosis was 78–86% and had favorable outcomes with an mRS of zero or one, 5–11% had independent outcomes with an mRS of two or three, 7–9% had very poor outcomes with an mRS of four or five, and 2–2.5% resulted in death [39]. In our cases, the percentage of an mRS of four or more cases was higher (13.6% vs. 9–11.5%), and the mortality rate was also higher (9.1% vs. 2–2.5%). Therefore, the prognosis for our group of cases is assumed to be relatively poor. The long-term prognosis for RCVS cases is determined by the occurrence of stroke [28]. The incidence of stroke in our cases was relatively higher than the incidence of COVID-19-unrelated RCVS (50.0% vs. 17%). Therefore, in the case of COVID-19-related RCVS, stroke is likely to be more common. This may be responsible for a poorer long-term prognosis. This is also supported by the result that our patients’ clinical prognoses were poorer than that of the COVID-19-unrelated RCVS patients.

### 4.7. Mechanism of COVID-19 Related RCVS

**Figure 3:**
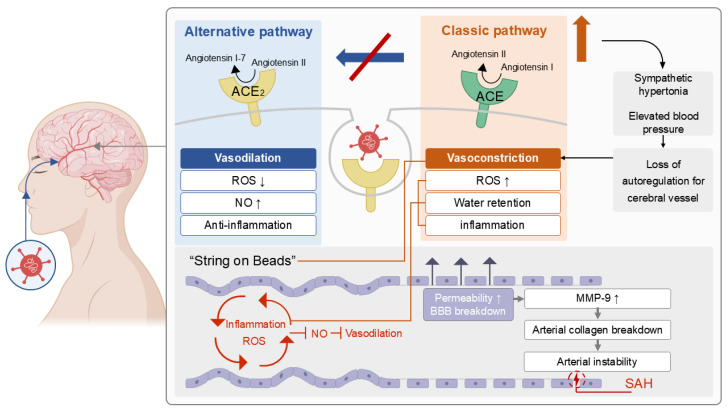
Mechanism of COVID-19-related reversible cerebral vasoconstriction syndrome during COVID-19 infection; viral elements invade endothelial cells through angiotensin-converting enzyme receptor 2. This can damage endothelial cells, resulting in microvascular dysfunction. Additionally, the angiotensin-converting enzyme 2 receptor is down-regulated during the virus invasion process, causing hyperactivation of the classical renin-angiotensin system pathway. This can cause sympathetic hypertonia in the cerebral vascular wall and a loss of the self-regulatory function of the cerebral vascular system due to a surge in blood pressure. Hyperactivation of the classic renin-angiotensin system pathway can also cause systemic inflammation and water retention; these can lead to increased blood–brain barrier permeability and the breakdown of arterial collagen, ultimately resulting in arterial instability. ACE = angiotensin-converting enzyme; ROS = reactive oxygen species; NO = nitrous oxide; BBB = blood–brain barrier; MMP-9 = matrix metalloproteinase-9.

Mechanistic details may be responsible for the relatively poor prognosis in COVID-19-related RCVS patients. First, respiratory symptoms related to COVID-19, such as coughing and sneezing, might act as triggers for RCVS. These recurring triggers could potentially accelerate the progression of RCVS. Second, COVID-19 has a mortality rate of around 1%. Compared to the COVID-19-unrelated RCVS cases, COVID-19 infection-related deaths could elevate mortality rates. The pathogenesis of COVID-19-related RCVS is not clearly understood, but the hypotheses raised are outlined in Figure 3.

One of these hypotheses states that, during COVID-19 infection, viral elements invade endothelial cells through ACE2 receptors. As the vascular endothelium plays an important role in regulating and maintaining vascular tone, microvascular dysfunction occurs as a result of endothelial cell destruction caused by COVID-19 infection [40,41,42]. In addition, the ACE2 receptor is down-regulated during the infection process, resulting in the hyperactivation of the classical renin–angiotensin–aldosterone system pathway and/or hypertension of the sympathetic nervous system in the blood–brain barrier (sympathetic hypertonia) [43]. As a result, complete or segmental vasoconstriction occurs, resulting in spikes in blood pressure. This may lead to a loss of autoregulation in cerebral vessels.

The hyperactivation of the classical renin–angiotensin–aldosterone system pathway also triggers systemic inflammation [44], and increased inflammation could increase the blood–brain barrier’s permeability [44]. The disruption of the blood–brain barrier can cause an increase in matrix metalloproteinase 9 level [44]. An increased level of matrix metalloproteinase 9 can cause a breakdown of arterial collagen, leading to arterial instability and subarachnoid hemorrhage [44,45]. Vasodilation and the loss of cerebral autoregulation can also occur due to the local inflammatory release of vasoactive substances.

**Figure 4:**
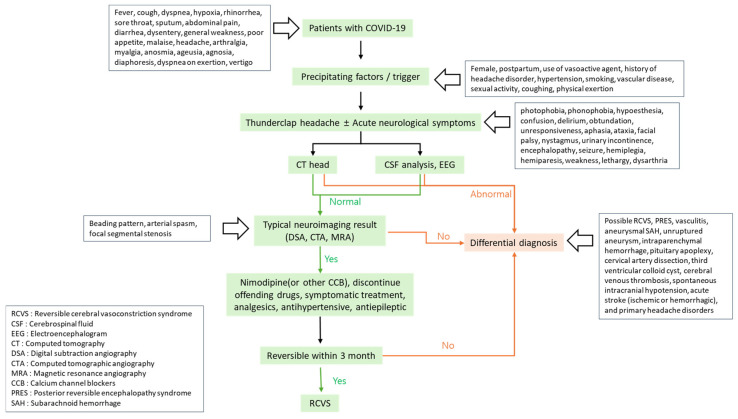
Diagnostic and management flowchart for RCVS in patients with COVID-19.

In addition to the proposed mechanisms outlined in Figure 3, the clinical course of COVID-19-related RCVS, from initial symptoms to diagnosis and management, is summarized in Figure 4. This flowchart visually illustrates the diagnostic steps for RCVS in patients with COVID-19.

### 4.8. Limitations

First, RCVS is rare, and COVID-19-related RCVS is even rarer, resulting in a small total number of cases. Although the difference in the number of cases is small, the percentage change appears to be larger. Second, we performed univariate and multivariable logistic regression analyses to compare the outcomes across the different subsets of cases (e.g., by age, sex, hypertension history, and the use of offending drugs). However, no statistically significant results were found, likely due to the small sample size. Third, vaccination status was known for only six of the twenty-four patients (one unvaccinated and five vaccinated with known dates and vaccine types). The vaccination status of the remaining 18 patients was unknown, preventing us from including their vaccination status in further analyses or assessing its potential impact on the outcomes. Fourth, data on cerebral vascular segment involvement during the onset of COVID-19-unrelated RCVS were unavailable, which limited our ability to determine the differences between COVID-19-related and COVID-19-unrelated RCVS segment involvement. Fifth, some cases lacked detailed follow-up data and long-term outcomes, making it difficult to fully assess the prognosis and long-term impact of RCVS in the context of COVID-19. This inconsistency in reporting follow-up data may limit the comprehensiveness of our prognosis assessment. Finally, most of the included cases originated from case reports. Since case reports mainly describe severe and unusual cases, our analysis may be biased.

## 5. Conclusions

In conclusion, our study described the clinical characteristics of COVID-19-related RCVS. While recovery from vasoconstriction, as determined by brain angiographic findings, is relatively common, our systematic review indicates a relatively poor neurological prognosis in COVID-19-related RCVS. It is crucial for clinicians to recognize the potential association between COVID-19 and RCVS, particularly in patients presenting with severe headaches or neurological symptoms following COVID-19 infection. Early diagnosis through timely neuroimaging and clinical evaluation is essential to improve the outcomes. Consequently, intensive care may be necessary for patients with COVID-19-related RCVS.

## Figures and Tables

**Table 1:** Demographic data, past history, and risk factors of the included cases.

		Age/Sex	Ethnicity	Past Medical History	Past Medication History	Vaccination Status	Initial Blood Pressure	Risk Factors Associated with RCVS	
								Medical History	Offending Drugs
1	Mansoor et al., 2021 [13]	31/F	N/A	spina-bifida, idiopathic thoracolumbar scoliosis (post hardware spinal fusion surgery 5 years ago)	X (comprehensive drug screen, including amphetamines, cocaine, nicotine, alcohol, and ecstasy were negative)	N/A	Normo-tensive		X
2	Dakay et al., 2020 [14]	35 */F	N/A	Migraines	N/A (no typical triggers for RCVS on review of medication)	N/A	N/A	Migraines	X (no typical triggers for RCVS upon review of medication)
3	Arandela et al., 2021 [15]	62/F	Caucasian	Hypertension, type 2 diabetes mellitus, migraine, obesity (BMI 37), obstructive sleep apnea syndrome	Duloxetine, citalopram	N/A	145/64	Migraine, Hypertension	Duloxetine, citalopram
4		39/F	Caucasian	Hypertension, Type 2 Diabetes Mellitus, Hyperlipidemia, Obesity (BMI 49)	Bupropion, fluoxetine	N/A	144/73	Hypertension	Bupropion, fluoxetine
5		47/F	African American	Hypertension, Type 2 Diabetes Mellitus, Hyperlipidemia, Obesity, Migraine	Citalopram	N/A	270/-	Hypertension, Migraine	Citalopram
6		55/M	Caucasian	X	Nasal decongestants	N/A	119/59	X	Nasal decongestants
7		35/M	Caucasian	X	? Supplements: Red rice yeast, milk thistle, fish oil, turmeric?	N/A	149/78		X
8		54/F	Caucasian	Hyperlipidemia	Citalopram, oxcarbazepine, ibuprofen, excedrin, cetrizine, denosumab, rosuvastatin, tamoxifen	N/A	148/88		Citalopram, oxcarbazepine, ibuprofen, excedrin, Tamoxifen
9		54/F	Haitain Creole	Hypertension, hyperlipidemia	X	N/A	218/123	Hypertension	X
10		37/F	African American	Tobacco use	Escitalopram, lurasidone, barbiturates, opiates	N/A	140/65	Tobacco use	Escitalopram, lurasidone, Barbiturates, opiates
11		25/F	Caucasian	Hypertension, obesity	Marijuana	N/A	138/92	Hypertension	Marijuana
12		21/M	African American	X	X	N/A	80/50	X	X
13	Srinivasan et al., 2021 [16]	18/M	African American	Asthma	9-THC (Cannabinoids, by urinary drug screen), not on medication that increase bleeding risk	N/A	149/79	X	9-THC (Cannabinoids, by urinary drug screen)
14	Harahsheh et al., 2022 [24]	44/M	N/A	Major depressive disorder	Intermittent cannabis use (tetrahydrocannabinols, urinary drug screen), duloxetine	N/A	195/126	X	Tetrahydrocannabinols, duloxetine
15	Sadeghizadeh et al., 2022 [25]	10/F	N/A	[Day 1] Admitted due to MIS-C left ventricular dysfunction [Day 4] ST elevation on electrocardiogram pancreatitis (confirmed on ultrasonogram)	[Day 1] Pulsed methylprednisolone (30 mg/kg, 2 dose), intravenous immunoglobulin (2 g/kg) followed by methylprednisolone (2 mg/kg) epinephrine, milrinone (due to hemodynamic instability and left ventricular dysfunction seen on ECG) [Day 4] Intravenous immunoglobulin (2 g/kg), levetiracetam dose of levetiracetam increased, and carbamazepine, gabapentin were added	N/A	N/A	MIS-C	IVIG, epinephrine, milrinone, levetiracetam, carbamazepine, gabapentin
16		6/M	N/A	Admitted due to MIS-C, Kawasaki shock syndrome (5 days prior)	Fluid therapy, epinephrine, milrinone (for treating shock) packed cells (because of low Hb), pulsed methylprednisolone (30 mg/kg, 2 doses), IVIG (2 g/kg) for 5 days	N/A	N/A	MIS-C, Kawasaki shock Syndrome	Epinephrine, milrinone, IVIG
17	Pedro et al., 2022 [26]	35/F	N/A	Episodic migraine with typical aura obesity, lower limb varicose veins, smoking (18 pack-years)	PRN sumatriptan progestin contraceptive implant	N/A	normotensive	migraine with typical aura, smoking (18 pack-years)	PRN sumatriptan, progestin contraceptive implant
18	Dutta et al., 2021 [27]	38/M	N/A	X (non-hypertensive, non-diabetic, non-smoker, teetotaler)	X (no history of medications intake or recreational drug use in the recent past)	X (no history of recent vaccination)	124/84	X	X
19	Sousa et al., 2023 [28]	30/F	N/A	X (no history of trauma or connective tissue disorder)	Corticosteroid (for COVID-19)	N/A	N/A	X	N/A (limited information about medication history except corticosteroid for COVID-19)
20	Our case	37/F	Asian	Episodic migraine	PRN acetaminophen or ibuprofen	Completion of booster shot (Pfizer, 3 months ago)	168/102	migraine	Acetaminophen, ibuprofen
21	Our case	48/F	Asian	Chronic migraine, depression, anxiety	Topiramate, benzodiazepine, mirtazapine	Completion of booster shot (Pfizer, 5 months ago)	210/102	migraine	Topiramate, benzodiazepine, mirtazapine
22	Our case	52/F	Asian	Hypertension, idiopathic stabbing headache	Amlopidine, telmisartan, gabapentin	Completion of booster shot (Pfizer, 4 months ago)	182/95	hypertension, idiopathic stabbing headache	Gabapentin
23	Our case	43/F	Asian	Episodic migraine	PRN zolmitriptan, acetaminophen or ibuprofen	Completion of booster shot (Pfizer, 6 months ago)	175/97	migraine	Zolmitriptan, acetaminophen, ibuprofen
24	Our case	33/F	Asian	Episodic migraine	PRN almotriptan, acetaminophen or ibuprofen	Completion of booster shot (Pfizer, 4 months ago)	165/102	migraine	Almotriptan, acetaminophen, ibuprofen

* Because patient #2’s age is described as “in her thirties”, she was assumed as 35 years old. RCVS = reversible cerebral vasoconstriction syndrome; MIS-C = multisystem inflammatory syndrome in children; IVIG = intravenous immune globulin; PRN = pro re nata.

**Table 2:** Clinical features of COVID-19-related RCVS.

#	Confirmatory Test for COVID-19 Diagnosis	Symptoms/Signs			Complications of RCVS	RCVS2 Score	Lab Findings and Other Test Results	Severity of COVID-19 (ICU)
		COVID-19-Related Symptoms	RCVS-Related Symptoms	ETC.				
1	RT-PCR(+); nasopharyngeal swab	Mild cough	Holocranial headaches, visual changes, nausea (1 day history)		X	6 *	coagulation profile: normal CSF analysis: normal (normal cell count, protein, viral(-), autoimmune Ab(-))	X
2	RT-PCR(+); nasopharyngeal swab	Severe cough (few weeks)	Severe thunderclap headache (3 days)		cSAH	10	Coagulation parameter: normal hCG (-)	O
3	PCR(+)	Dyspnea on exertion, hypoxia, malaise, arthralgias, posterior headache, poor appetite, diarrhea, generalized weakness	New posterior headache, hyperactive delirium		X	4 †	Increased above normal levels: PT, D-dimer, CRP, LDH Decreased below normal levels: CK	O
4	PCR(+)	Asymptomatic	Thunderclap headache, confusion, right hemiparesis		SAH, parenchymal hemorrhage, edema, acute ischemic change	10	↑: WBC, PT, CRP	X (for SAH/vasculopathy)
5	PCR(+)	Hypoxia, dyspnea, fever	Obtundation, encephalopathy		cSAH, gyral swelling	3	↑: WBC, fibrinogen, D-dimer, ferritin, CRP, ESR, procalcitonin	O
6	PCR(+)	Severe dyspnea and hypoxia (severe ARDS) requiring intubation	Broca’s aphasia (difficulty speaking), weakness, right hemiparesis, left arm hemiparesis		X	3	↑: WBC, neutrophils, PT, D-dimer, ferritin, CRP, LDH, procalcitonin	O
7	PCR(+)	Dyspnea	Thunderclap headache (recurrent, 3 episodes, 2 days between episodes)		X	5	↑: WBC	X
8	PCR(+)	Mild cough, sore throat	Thunderclap headache (recurrent, 2 weeks between episodes)		cSAH, SDH	10	↑: neutrophils ↓: PT	X
9	positive test	Cough, diaphoresis, dyspnea	Encephalopathy, aphasia, right hemiplegia		Recent infarction, sulcal effacement	1	↑: PT, fibrinogen, D0dimer, ferritin, CRP, ESR, LDH ↓: CK	O
10	positive test	Fever, myalgia, cough, headache	Thunderclap headache (recurrent, 3 episodes)		Intracerebral hemorrhage, recent ischemic infarct	9	↑: neutrophils, PT, D-dimer, CRP, ESR	X
11	PCR(+)	Headache, anosmia, agnosia	Thunderclap headache(recurrent)		Ischemic stroke	9	↓: neutrophils	X
12	PCR(+)	Severe dyspnea, hypoxia (severe ARDS)	Obtundation, encephalopathy	Acute cardiogenic shock, HFrEF 10%, acute liver/pancreas/ respiratory/renal failure	X	0	↑: neutrophils, D-dimer ↓: WBC, fibrinogen	O
13	positive (RCVS in the setting of COVID-19 infection)	Asymptomatic	Thunderclap headache, nausea, vomiting (for the past 2 days before admission) unresponsiveness (GCS 3)		Non-aneurysmal SAH, ICH	9	Blood test: normal (PLT count, coagulation panel, CRP) Vasculitis evaluation: normal (ANA, CRP, SED rate, ANCA, serum neurosyphilis Antibody)	X (for GCS 3, SAH, not for COVID-19)
14	RT-PCR(+); nasopharyngeal swab	Anosmia, ageusia mild upper respiratory symptom	Severe bifrontal headache (present intermittently and has been progressive despite symptomatic treatment with simple analgesics)	Persistently elevated BP (due to right renal mass; pleomorphic sarcoma) Acute onset of language difficulty (moderate to severe expressive aphasia) Encephalopathy, expressive aphasia (due to hypenatremia, persistent elevation in BP)	X	8	↑Cr (1.58 mg/dL), hyponatremia (129 mmol/L)	X (for hypo-natremia, agitation, not for COVID-19)
15	X (contact with COVID-19 patients 1 week prior to admission)	[Day 1] fever, dysentery	[Day 4] Abrupt severe frontal thunderclap headache (resolved around day 25), nausea, vomiting, seizure (suspected) with upward gaze, urinary incontinence, diplopia, visual impairment (blurred vision), proximal weakness of lower extremity, seizure, photophobia, hemiplegia	[Day 4] Epigastric pain, chest pain	[Day 8] Multifocal infarcts [5 months F/U] Chronic infarcts	> 5 (9 *) †	[Day 1] Blood test: cytopenia, ↑troponin, ↑inflammatory marker ECG: left ventricular dysfunction [Day 4] Blood test: ↑ferritin, ↑troponin, ↑amylase, ↑lipase ECG: ST elevation	X
16	Serum IgG(+)	[Day 1] Fever, diarrhea, severe abdominal pain	[Day 5] Thunderclap headache (resolved around Day 9–10), nausea, vomiting, photophobia	[Day 11] Diagnosed as nephritis : hematuria, proteinuria, dysmorphic RBC (60%), hypertension	X	>5 (8 *) †	↑ inflammatory markers, low hemoglobin ECG: myocardial dysfunction	X
17	RT-PCR(+); nasopharyngeal swab	Asymptomatic (no symptoms were present during the examination)	Two episodes of bilateral occipital thunderclap headache (3 h interval), associated with nausea, vomiting, photophobia, phonophobia. pain intensity 10/10, did not improve after taking ibuprofen. Lethargy, gaze-evoked nystagmus, horizontal hypermetric saccades, left facial hypoesthesia, mild facial palsy, dysarthria, right sided limb ataxia	Did not tolerate orthostatic position, straining, lifting heavy objects	Cerebellar ischemia	7	Blood count, C-reactive protein, cardiac markers, coagulation studies: unremarkable transthoracic ECG: unremarkable	X
18	RT-PCR(+); oropharyngeal and nasopharyngeal swabs	Asymptomatic (no history of fever, cough, sore throat, diarrhea, loss of smell or taste, trauma)	Mild holocranial headache and confusion (for last 2 days)		X	−2 *	Inflammatory markers: within normal limits ABG, CSF analysis: normal EEG: diffuse theta waves in the background Coagulation profile: normal	X
19	RT-PCR(+)	Respiratory symptoms	Thunderclap headache, right hemiplegia, decreased consciousness		ICH	6 *	N/A	X
20	PCR(+)	Fever, sore throat, general weakness	Thunderclap headache, nausea, vomiting		X	6	CSF: normal vasculitis lab, autoimmune lab: all negative EEG (day 8): normal Hemoglobin 10.4 Thyroid function test: normal	X, no supplemental O^2^ requirement
21	PCR(+)	Fever, sore throat, general weakness, cough, rhinorrhea	Thunderclap headache, visual disturbance		PRES	6	Vasculitis lab, autoimmune lab: all negative EEG (day 8): mild cerebral dysfunction Hemoglobin 9.8 Thyroid function test: normal	X, no supplemental O^2^ requirement
22	PCR(+)	Fever, sore throat, diarrhea	Thunderclap headache, nausea, vomiting		X	6	CSF: normal vasculitis lab, autoimmune lab: all negative EEG (day 8): normal Thyroid function test: normal	X, no supplemental O^2^ requirement
23	PCR(+)	Fever, sore throat, general weakness	Thunderclap headache, confusion, nausea, vomiting		PRES, cortical SAH	9	Vasculitis lab, autoimmune lab: all negative EEG (day 8): normal Thyroid function test: normal	X, no supplemental O^2^ requirement
24	PCR(+)	Fever, cough, sputum, general weakness, vertigo	More severe migraineous attack (visual analogus scale 5 → 10), migrainous status (more than 72 h, nausea, vomiting)		X	9	CSF: normal Vasculitis lab, autoimmune lab: all negative EEG (day 8): normal Thyroid function test: normal	X, no supplemental O^2^ requirement

*† calculated value is indicated with * next to the score and marked with † if it did not fit the age category. PCR = polymerase chain reaction; ARDS = acute respiratory distress syndrome; GCS = Glasgow coma scale; VAS = visual analog scale; BP = blood pressure; CSF = cerebrospinal fluid; EEG = electro encephalography; ECG = electrocardiogram; ABG = arterial blood gas; PRES = posterior reversible encephalopathy syndrome; SAH = subarachnoid hemorrhage; SDH = subdural hemorrhage; ICH = intracranial hemorrhage; ACA = anterior cerebral artery; HFrEF = heart failure with reduced ejection fraction.

**Table 3:** Treatment, imaging findings, and prognosis.

#	Treatment		Radiologic Findings During Hospitalization			Outcomes on Discharge	mRS	Follow Up Period	Follow Up Data for Neuroimaging and Prognosis
	Treatment for RCVS	Treatment for Other Medical Conditions	Brain CT/MRI	Brain CTA, MRA, DSA	Findings in Other Imaging Modalities				
1	PO nimodipine 60 mg Q4 h for 21 days Aspirin 81 mg for 21 days		CT: normal (did not show signs of ICH or ischemia) MRI: bilateral patchy gyral pattern of T2-FLAIR hyperintensities (predominantly parieto-occipital, frontal lobes)	CTA: beading pattern (basilar a.) CT-Venogram: normal (no venous sinus thrombosis) DSA (7 days later): resolution of basilar arteriospasm		Symptoms resolved	0 *	N/A	N/A
2	Verapamil, aspirin, analgesics	Levetiracetam	CT: bilateral high frontal cSAH	DSA, CTA (on admission): left vertebral a. dissection (V2 segment) DSA (Day 7): bilateral mild to moderate anterior circulation vasospasm		Discharged home with verapamil and follow up CTA	0 *	N/A	N/A
3	IV dexamethasone		MRI: Hyperintense T2 and T2 FLAIR signal (frontal, temporal, parietal, occipital subcortical white matter, and pons) without restricted diffusion or enhancement.	MRA: Long segment irregularity and narrowing of the distal left MCA with the most pronounced involvement of the ACA and left MCA branches. Long segment irregularity and narrowing of distal right PCA branches with lesser involvement of the distal left PCA branches.		Discharged to acute rehab; mRS 0	0	3 Months	MRI/MRA (3 months follow up): interval resolution of previously seen predominantly white matter T2 FLAIR hyperintensity in the parietal and occipital lobes involving subcortical and deep white matter Central pontine T2 FLAIR hyperintensity persists and is not substantially changed in the interval. There was interval resolution of the previously seen irregularity involving the distal left MCA, left ACA, right PCA, and left PCA.
4	IV dexamethasone		CT: Acute parenchymal hemorrhage centered in the left frontal lobe measuring 2.8 × 2.4 × 2.3 cm with adjacent vasogenic edema. There is a large volume SAH that emanates from this position and is likely a direct extension from the parenchymal hemorrhage. It is also present in the interpeduncular cistern, ambient cistern, and extends to the left sylvian fissure. There is additional SAH present in the left frontoparietal convexity and right temporal sulci.	CTA: Left MCA M2 distribution multivessel long segment high-grade stenosis affecting essentially all M2 segment branches. High-grade stenosis extends into the M3 segment and M4 segment of multiple of these branches as well, in the region of the large SAH. Low attenuation in the left thalamus and medial left basal ganglia is non-specific but suspicious for acute ischemic change. DSA: Multifocal caliber irregularities in the left MCA, affecting the larger proximal branches as well as the smaller distal vessels.		Discharged home with home health; mRS 2	2	3 Months	CTA (3 months follow up): significant interval improvement of previously seen stenosis involving the left M2, M3, and M4 segments
5	PO prednisone		MRI: Large areas of restricted diffusion involving the right MCA and left parietal; also, FLAIR gyral swelling in the right posterior cerebral cortex with minimal convexal SAH of the right parietal lobe	CTA (1): Approximately 50% stenosis of the proximal portion of the left internal carotid artery. Multifocal areas of moderate to marked stenosis of nearly all intracranial vessels. Vertebrobasilar junction and the basilar artery demonstrate multifocal areas of stenosis and luminal irregularity but are patent throughout its course. There is a luminal irregularity near the tip of the basal artery at the takeoff of the bilateral PCA. CTA (2): Moderate irregular stenosis of the distal left internal carotid artery and proximal portions of the left MCA and ACA. Severe stenosis of the terminal right ICA and of the proximal right ACA and MCA. The more distal right MCA and its branches are partially patent, improved from prior.		Deceased	6 *	N/A	N/A
6	IV dexamethasone			CTA: Diminutive left MCA (M1) branch with only minimal reconstitution of a few distal cortical branches. Paucity of opacified intracranial arteries with focal narrowing. Occlusion of mid to distal right ACA A2 segment,		Currently admitted, remained intubated on ventilator	N/A	N/A	N/A
7	X		MRI/MRA: unremarkable	CTA: unremarkable MRI/MRA: unremarkable DSA: unremarkable		Discharged home; mRS 0	0	N/A	N/A
8	X		MRI: cSAH of left superior parietal lobe, small SDH of left parietal lobe	CTA: cSAH left superior parietal lobe, subtle multifocal stenosis of the bilateral ACA and MCA DSA: Mild left cervical ICA irregularity		Discharged home; mRS 0	0	3 weeks	CT/MRI/MRA (3 weeks follow up): CT head with residual left parietal SAH; MRI/MRA with residual SAH, small SDH
9	X		MRI: Mixed signal intensity areas corresponding to the region of hypodensity and sulcal effacement in the left frontal lobe and insula are consistent with recent infarction. Areas of restricted diffusion in other portions of the left frontal, parietal, and occipital lobes, as well as left posterior limb of internal capsule extending to brainstem and punctate area in the right frontal lobe. Moderate bilateral confluent FLAIR hyperintensities are typical of small vessel disease.	CTA: left inferior M2 occlusion DSA: left inferior division M2 occlusion s/p mechanical thrombectomy with TICI 3 reperfusion. Diffuse vasculopathy of the M2 and M3 divisions of bilateral MCA, left pericallosal artery.		Discharged to acute rehab	3	N/A	
10	PO nimodipine, IA verapamil, IA milrinone IV methyl-prednisolone		MRI/MRA: Hemorrhage of 3 cm in the left occipital lobe. Narrowing of the precavernous left ICA. Slightly decreased caliber of the M1 segment of the left MCA is seen; however, no significant focal stenosis is seen. The M1 segment of the right MCA is unremarkable. ACA and PCA are unremarkable.	CTA (1): Progressive moderate/severe narrowing involving the proximal/mid M1 segment of the left MCA and distal M1 of right MCA. Multifocal severe luminal narrowing of M2 branches of bilateral MCA. Moderate stenosis of right A1 and right P1. MRI/MRA: hemorrhage of 3 cm in the left occipital lobe. Narrowing of the precavernous left ICA. Slightly decreased caliber of the M1 segment of the left MCA is seen, however no significant focal stenosis is seen. The M1 segment of the right MCA is unremarkable. ACA and PCA are unremarkable. CTA (2): Stable IPH in the left occipital lobe, more extensive than better demarcated hypodensities in the bilateral parietal and occipital lobes representing recent ischemic infarcts; multifocal mod/severe involving bilateral M1/M2 segments, bilateral A1 segments, right > left, and bilateral A2, bilateral PCA which worsened from prior condition. DSA: Diffuse multifocal narrowing of the intracranial circulation involving the MCA, ACA, and PCA as well as the PICA/AICA. There was a moderate improvement after the injection of a small dose of milrinone and verapamil.		Deceased	6 *	N/A	
11	IA nicardipine		MRI/MRA: Acute ischemic bilateral occipital strokes, diffuse vasospasm	CTA: diffuse vasospasm MRI/MRA: acute ischemic bilateral occipital strokes, diffuse vasospasm DSA: Subtle diffuse irregularity of the distal MCA territories bilaterally. Attenuated PCA parietal occipital territories.		Discharged home with vision rehab	2	N/A	N/A
12	IV dexamethasone			CTA: Diffuse intracranial vascular abnormalities concerning vasospasm or vasculitis. No large territory infarct on CT perfusion.		Currently admitted, extubated in ICU, to be downgraded soon	N/A	N/A	N/A
13	Emergent right decompressive craniectomy IV verapamil (80 mg, every 8 h)		CT (at admission): normal (no acute intracranial abnormality) CT (4h after admission): hemorrhage with 66 cc (6.4 × 4.4 × 4 cm) in the right frontal lobe with 7mm of leftward midline shift, mass effect on the right lateral ventricle, and SAH in the right frontal and temporal lobes. MRI (post decompression): SAH in the right frontal and parietal lobe.	CTA: multifocal areas of stenosis without large vessel occlusion: Bilateral MCA (M2/M3); Bilateral PCA (P2/P3); Right ACA (A2). DSA: mild to moderate diffuse focal intracranial stenosis concerning RCVS Cerebral angiogram (Day 13): near-complete resolution of the previously seen diffuse focal stenosis		Transferred to an inpatient physical rehabilitation facility (2 weeks)	0 *	3 months	Full functional recovery over the course of the following 3 months
14	nicardipine drip then oral nifedipine (for BP control)	Right nephrectomy with tumor resection; pleomorphic sarcoma	head CT: normal (negative for acute intracranial abnormalities)	CTA: large vessel occlusion (-), focal narrowing in the following: - Left PCA (near the P2-P3 junction); - Left MCA (beyond the origin of the superior division); subtle narrowing of the - Right MCA (proximal M1 segment). MRA (few days later): resolution of intracranial vessel narrowing after BP control was achieved	Renal ultrasound: heterogenous mass infiltrating the right kidney with invasion into the adjacent liver Abdomen, pelvis CT (IV contrast): hypoenhancing right renal mass (18 cm diameter) that was replacing the right kidney with intrahepatic invasion along the subcapsular surface of the liver	Free of headaches	0 *	N/A	N/A
15		[Day 8] Methylprednisolone pulse, single dose of infliximab (to control inflammation)	MRI (Day 8): multifocal infarcts	MRA (Day 8): widespread multifocal foci of arterial narrowing bilaterally - estimated by figure : Left A2, M1, M2/Right A2, M2		[Day 49] Discharged in good general condition with fludrocortisone, aspirin, and oral prednisolone (2 mg/kg).	0 *	5 months	Two month: no residual neurologic deficit MRI, MRA (5 months): residual smalll foci of chronic infarct and resolution of the areas of vasoconstriction
16		Third pulse methylprednisolone therapy (30 mg/kg) (for treating nephritis)	MRI (Day 6): no infarcts	MRA (Day 6): multifocal bilateral foci of arterial vasoconstriction - estimated by figure : bilateral ACA, MCA, PCA		[Day 22] Discharged in good general condition, with prednisolone (2 mg/kg), aspirin, and amlodipine.	0 *	6 Months	One month: no neurological deficits MRI, MRA (6 months): complete resolution of vasoconstriction
17	PO nimodipine 30 mg 3 times/day titrated to half of the dosage on day 7 (due to improvement of cerebral blood flow velocities after repeating TCD) Antiplatelet therapy during the hospital stay		CT (at admission): normal CT (8 h later): decreased attenuation of the right cerebellar hemisphere	CTA (8 h later): occlusion of proximal segment of right SCA MRA: stenosis of the prepontine segment and fusiform dilatation of the ambient and quadrigeminal segments of the right SCA, eccentric T1 weighted hyperintensity suggestive of arterial dissection with intramural thrombus. Luminal irregularities in the V4 segment of both vertebral arteries, basilar artery trunk, AICA, left SCA, PCA, bilateral A2, and M2.	Transcranial doppler ultrasound: increased velocity at the A1, left M1, basilar a., and bilateral PCA.	Discharged to a rehabilitation facility with only mild right-sided ataxia and dysarthria.	1 *	N/A	N/A
18	PO nimodipine 60 mg 4 times/day Simple analgesics when required for headache		Contrast (gadolinium)-MRI of brain: did not reveal any abnormality or evidence of venous sinus thrombosis.	Contrast MR-venogram: did not reveal any abnormality or evidence of venous sinus thrombosis. CTA, DSA: narrowing of the terminal parts of both ICA and bilateral MCA with pruning of distal flow - estimated by figure: bilateral M4 DSA, CTA (after 14 days): near total improvement of the caliber of bilateral terminal ICA and bilateral MCA with near normalization of distal flow.	High resolution CT of thorax: did not reveal any abnormality.	Patient had significant improvement of the symptoms within the next few days.	0 *	N/A	N/A
19	Decompressive craniotomy for intracerebral hemorrhage		CT (6 days after being diagnosed with COVID-19): large left parenchymal intracerebral hemorrhage	DSA: Segmental narrowing followed by dilations in V2, V3 segments of the right and left vertebral arteries, suggesting bilateral vertebral artery dissection. Mild segmental narrowing followed by a normal appearance in both the left PICA and distal branches of the left MCA.	Chest CT: lung parenchymal involvement of 30–50%, suggestive of viral pneumonia	Poor outcome; classified with mRS 5 at discharge	5	N/A	N/A
20	PO nimodipine 60 mg Q4 h (28 days) amlodipine, fimasartan (for BP control)		CT: unremarkable MRI: unremarkable	MRA (Day 8): multifocal mild to moderate stenosis MCA M2, ACA A2 portion multifocal moderate stenosis bilateral PCA P2 portion		Free of headaches; neurologically fully recovered	0	6 months	No recurrence, completely improved MRA (3 months): fully recovered
21	PO nimodipine 60 mg Q4 h (90 days), aspirin 100 mg daily IV nicardipine for BP control amlodipine, fimasartan (for BP control)	rosuvastatin 10 mg daily	MRI: Hyperintense T2 and T2 FLAIR signal bilateral occipital subcortical white matter without restricted diffusion or enhancement.	MRA (Day 9): multifocal mild stenosis MCA M2, ACA A2 multifocal severe stenosis bilateral PCA P2 portion		Free of headaches; mild visual field defect (Rt quardrianopsia) remained	1	8 months	MRA (3 months): bilateral PCA mild stenosis (improved but not fully recovered); left occipital lobe with high signal partially recovered,
22	PO nimodipine 60 mg Q4 h (28 days) amlodipine, fimasartan (for BP control)	remdesivir (for COVID-19)	CT: unremarkable MRI: unremarkable	MRA (Day 8): multifocal moderate stenosis bilateral PCA P1, P2 portion		Free of headaches; neurologically fully recovered (completely improved)	0	6 months	MRA (4 months): fully recovered
23	PO nimodipine 60 mg Q4 h (90 days) IV nicardipine (for BP control) amlodipine, fimasartan (for BP control)	atorvastatin 40 mg daily	MRI: cortical SAH of right occipital lobe MRI: hyperintense T2 and T2 FLAIR signal bilateral occipital subcortical white matter without restricted diffusion or enhancement.	MRA (Day 9): multifocal mild stenosis MCA M2, ACA A2 multifocal severe stenosis bilateral PCA P1, P2 portion		Free of headaches; neurologically fully recovered (completely improved)	0	8 months	MRA (5 months): mild Rt M2 stenosis, mild right P2 stenosis, and Lt fully recovered
24	PO nimodipine 60 mg Q4 h (28 days) amlodipine, fimasartan (for BP control)		CT: unremarkable MRI: unremarkable	MRA (Day 8): multifocal moderate stenosis bilateral ACA (A2, MCA M2, PCA P1, P2 portion)		Free of headaches; neurologically fully recovered (completely improved)	0	6 months	MRA (3 months): fully recovered

* Calculated value based on clinical descriptions in the article when not explicitly stated. SAH = subarachnoid hemorrhage; DSA = digital subtraction angiography; TICI = thrombolysis in cerebral infarction; ACA = anterior cerebral artery; MCA = middle cerebral artery; PCA = posterior cerebral artery; SCA = superior cerebellar artery; AICA = anterior inferior cerebellar artery; PICA = posterior inferior cerebellar artery; ICA = intracranial artery.

**Table 4:** Association between patient characteristics and risk factors with ICU admission.

	Univariable			Multivariable		
	OR	95% CI	*p*	OR	95% CI	*p*
Age	1.08	1.00–1.20	0.100	1.15	1.00–1.48	0.123
Sex	0.77	0.11–6.82	0.796	0.06	0.00–3.46	0.256
Presence of complications	0.80	0.12–5.39	0.813	7.17	0.19–2041	0.383
History of hypertension	5.00	0.66–42.70	0.119	17.20	0.54–2158	0.154
Use of offending drug	0.29	0.04–2.05	0.208	0.03	0.00–0.916	0.111

## Data Availability

The data used in this study are available in PubMed (MEDLINE), SCOPUS, and Web of Science.

## Supplementary Materials

The following supporting information can be downloaded at the following website: https://www.mdpi.com/article/10.3390/jcm14020487/s1, Supplementary File S1: Table S1: Search terms for PubMed, SCOPUS, and Web of Science; Table S2: Cases excluded due to insufficient evidence for RCVS diagnosis; Table S3: Offending drugs of RCVS; Table S4: COVID-19-related symptoms; Table S5: RCVS-related symptoms; Table S6: Involved cerebral vascular segments; Table S7: Odds ratio analysis for mortality in RCVS; Table S8: Odds ratio analysis for complication in RCVS. Refs. [17,46,47,48,49,50,51,52,53,54,55,56,57] are cited in Supplementary Materials.

## References

[1] Alimohamadi Y., Sepandi M., Taghdir M., Hosamirudsari H. (2020). Determine the most common clinical symptoms in COVID-19 patients: A systematic review and meta-analysis. J. Prev. Med. Hyg..

[2] Ahmad I., Rathore F.A. (2020). Neurological manifestations and complications of COVID-19: A literature review. J. Clin. Neurosci..

[3] Bridwell R., Long B., Gottlieb M. (2020). Neurologic complications of COVID-19. Am. J. Emerg. Med..

[4] Calabrese L.H., Dodick D.W., Schwedt T.J., Singhal A.B. (2007). Narrative review: Reversible cerebral vasoconstriction syndromes. Ann. Intern. Med..

[5] Headache Classification Subcommittee of the International Headache Society (2004). The International Classification of Headache Disorders: 2nd edition. Cephalalgia.

[6] Harapan B.N., Yoo H.J. (2021). Neurological symptoms, manifestations, and complications associated with severe acute respiratory syndrome coronavirus 2 (SARS-CoV-2) and coronavirus disease 19 (COVID-19). J. Neurol..

[7] Topcuoglu M.A., Singhal A.B. (2016). Hemorrhagic Reversible Cerebral Vasoconstriction Syndrome: Features and Mechanisms. Stroke.

[8] Ducros A. (2012). Reversible cerebral vasoconstriction syndrome. Lancet Neurol..

[9] Sattar A., Manousakis G., Jensen M.B. (2010). Systematic review of reversible cerebral vasoconstriction syndrome. Expert Rev. Cardiovasc. Ther..

[10] Spadaro A., Scott K.R., Koyfman A., Long B. (2021). Reversible cerebral vasoconstriction syndrome: A narrative review for emergency clinicians. Am. J. Emerg. Med..

[11] Desai A.D., Lavelle M., Boursiquot B.C., Wan E.Y. (2022). Long-term complications of COVID-19. Am. J. Physiol. Cell Physiol..

[12] Lee M.J., Cha J., Choi H.A., Woo S.Y., Kim S., Wang S.J., Chung C.S. (2017). Blood-brain barrier breakdown in reversible cerebral vasoconstriction syndrome: Implications for pathophysiology and diagnosis. Ann. Neurol..

[13] Mansoor T., Alsarah A.A., Mousavi H., Khader Eliyas J., Girotra T., Hussein O. (2021). COVID-19 Associated Reversible Cerebral Vasoconstriction Syndrome Successfully Treated with Nimodipine and Aspirin. J. Stroke Cerebrovasc. Dis..

[14] Dakay K., Kaur G., Gulko E., Santarelli J., Bowers C., Mayer S.A., Gandhi C.D., Al-Mufti F. (2020). Reversible cerebral vasoconstriction syndrome and dissection in the setting of COVID-19 infection. J. Stroke Cerebrovasc. Dis..

[15] Arandela K., Samudrala S., Abdalkader M., Anand P., Daneshmand A., Dasenbrock H., Nguyen T., Ong C., Takahashi C., Shulman J. (2021). Reversible Cerebral Vasoconstriction Syndrome in Patients with Coronavirus Disease: A Multicenter Case Series. J. Stroke Cerebrovasc. Dis..

[16] Srinivasan A., Wilson B.C., Bear M., Hasan A., Ezzeldin O., Alim S., Elfallal S., Fang X., Ezzeldin M. (2021). Intracerebral Hemorrhage and Reversible Cerebral Vasoconstriction Syndrome in a Patient With COVID-19. Cureus.

[17] Song T.-J., Lee K.H., Li H., Kim J.Y., Chang K., Kim S.H., Han K.H., Kim B.Y., Kronbichler A., Ducros A. (2021). Reversible cerebral vasoconstriction syndrome: A comprehensive systematic review. Eur. Rev. Med. Pharmacol. Sci..

[18] Durrleman C., Naggara O., Grevent D., Belot A., Desgranges M., Boyer O., Chabrier S., Bader-Meunier B., Kossorotoff M. (2019). Reversible cerebral vasoconstriction syndrome in paediatric patients with systemic lupus erythematosus: Implications for management. Dev. Med. Child. Neurol..

[19] Uchida Y., Matsukawa N., Oguri T., Sakurai K., Miura T., Iwagaitsu S., Naniwa T., Ojika K. (2011). Reversible cerebral vasoconstriction syndrome in a patient with Takayasu’s arteritis. Intern. Med..

[20] Rocha E.A., Topcuoglu M.A., Silva G.S., Singhal A.B. (2019). RCVS(2) score and diagnostic approach for reversible cerebral vasoconstriction syndrome. Neurology.

[21] Ray S., Kamath V.V., Raju P.A., Kn R., N S. (2022). Fulminant Reversible Cerebral Vasoconstriction Syndrome in Breakthrough COVID 19 Infection. J. Stroke Cerebrovasc. Dis..

[22] Hmaidan S., Nguyen L., Johnson A. (2023). A Rare Case Report of Acute Neurologic Sequelae in a Young Primigravida With Recent COVID Pneumonia. Cureus.

[23] Scheer M., Harder A., Wagner S., Ibe R., Prell J., Scheller C., Strauss C., Simmermacher S. (2022). Case report of a fulminant non-aneurysmal convexity subarachnoid hemorrhage after COVID-19. Interdiscip. Neurosurg..

[24] Harahsheh E., Gritsch D., Mbonde A., Apolinario M., Hoxworth J.M., Demaerschalk B.M. (2022). Reversible Cerebral Vasoconstriction Syndrome in the Setting of COVID-19 and Pleomorphic Sarcoma: A Case Report. Neurologist.

[25] Sadeghizadeh A., Pourmoghaddas Z., Zandifar A., Tara S.Z., Rahimi H., Saleh R., Ramezani S., Ghazavi M., Yaghini O., Hosseini N. (2022). Reversible Cerebral Vasoconstriction Syndrome and Multisystem Inflammatory Syndrome in Children with COVID-19. Pediatr. Neurol..

[26] Pedro T., Maia R., Costa R., Martins B., Ribeiro J., Domingues R., Brito T., Abreu P., Castro P., Soares-dos-Reis R. (2022). Superior Cerebellar Artery Dissection in a Patient Diagnosed with Reversible Cerebral Vasoconstriction Syndrome: A Case Report. Sinapse.

[27] Dutta A., Chandra A., Gupta S., Ray B.K., Das D., Kumar R. (2021). Reversible cerebral vasoconstriction syndrome in a patient with COVID-19. Neurol. Asia.

[28] Sousa I.A., Neto E., Ricarte I.F., Pontes-Neto O.M. (2023). Reversible cerebral vasoconstriction syndrome associated with Chikungunya infection. BMJ Case Rep..

[29] Chen S.-P., Fuh J.-L., Wang S.-J., Chang F.-C., Lirng J.-F., Fang Y.-C., Shia B.-C., Wu J.-C. (2010). Magnetic resonance angiography in reversible cerebral vasoconstriction syndromes. Ann. Neurol..

[30] Ducros A., Boukobza M., Porcher R., Sarov M., Valade D., Bousser M.-G. (2007). The clinical and radiological spectrum of reversible cerebral vasoconstriction syndrome. A prospective series of 67 patients. Brain.

[31] Singhal A.B., Hajj-Ali R.A., Topcuoglu M.A., Fok J., Bena J., Yang D., Calabrese L.H. (2011). Reversible Cerebral Vasoconstriction Syndromes: Analysis of 139 Cases. Arch. Neurol..

[32] Rokkas T. (2020). Gastrointestinal involvement in COVID-19: A systematic review and meta-analysis. Ann. Gastroenterol..

[33] Alene M., Yismaw L., Assemie M.A., Ketema D.B., Gietaneh W., Birhan T.Y. (2021). Serial interval and incubation period of COVID-19: A systematic review and meta-analysis. BMC Infect. Dis..

[34] Quesada J.A., López-Pineda A., Gil-Guillén V.F., Arriero-Marín J.M., Gutiérrez F., Carratala-Munuera C. (2021). Incubation period of COVID-19: A systematic review and meta-analysis. Rev. Clin. Esp. (Engl. Ed.).

[35] Iba T., Levy J.H., Levi M., Thachil J. (2020). Coagulopathy in COVID-19. J. Thromb. Haemost..

[36] Savić D., Alsheikh T.M., Alhaj A.K., Lazovic L., Alsarraf L., Bosnjakovic P., Yousef W. (2020). Ruptured cerebral pseudoaneurysm in an adolescent as an early onset of COVID-19 infection: Case report. Acta Neurochir..

[37] Krasemann S., Haferkamp U., Pfefferle S., Woo M.S., Heinrich F., Schweizer M., Appelt-Menzel A., Cubukova A., Barenberg J., Leu J. (2022). The blood-brain barrier is dysregulated in COVID-19 and serves as a CNS entry route for SARS-CoV-2. Stem Cell Rep..

[38] Wang Z., Yang Y., Liang X., Gao B., Liu M., Li W., Chen Z., Wang Z. (2020). COVID-19 Associated Ischemic Stroke and Hemorrhagic Stroke: Incidence, Potential Pathological Mechanism, and Management. Front. Neurol..

[39] Varga Z., Flammer A.J., Steiger P., Haberecker M., Andermatt R., Zinkernagel A.S., Mehra M.R., Schuepbach R.A., Ruschitzka F., Moch H. (2020). Endothelial cell infection and endotheliitis in COVID-19. Lancet.

[40] Vieira C., Nery L., Martins L., Jabour L., Dias R., Simões E.S.A.C. (2021). Downregulation of Membrane-bound Angiotensin Converting Enzyme 2 (ACE2) Receptor has a Pivotal Role in COVID-19 Immunopathology. Curr. Drug Targets.

[41] Panther E.J., Lucke-Wold B. (2022). Subarachnoid hemorrhage: Management considerations for COVID-19. Explor. Neuroprotective Ther..

[42] Cezar-Junior A.B., Faquini I.V., Silva J.L.J., de Carvalho Junior E.V., Lemos L., Freire Filho J.B.M., de Lira Filho H.T., Pontes E.C.A., Almeida N.S., Azevedo-Filho H.R.C. (2020). Subarachnoid hemorrhage and COVID-19: Association or coincidence?. Medicine.

[43] Ursell M.R., Marras C.L., Farb R., Rowed D.W., Black S.E., Perry J.R. (1998). Recurrent intracranial hemorrhage due to postpartum cerebral angiopathy: Implications for management. Stroke.

[44] Poyiadji N., Shahin G., Noujaim D., Stone M., Patel S., Griffith B. (2020). COVID-19-associated Acute Hemorrhagic Necrotizing Encephalopathy: Imaging Features. Radiology.

[45] Yeo S.J., Kim S.J., Kim J.H., Lee H.J., Kook Y.H. (1999). Influenza A virus infection modulates the expression of type IV collagenase in epithelial cells. Arch. Virol..

[46] Bonura A., Iaccarino G., Rossi S.S., Capone F., Motolese F., Calandrelli R., Di Lazzaro V., Pilato F. (2023). Posterior reversible encephalopathy syndrome and reversible cerebral vasoconstriction syndrome in patients with COVID-19 infection: Is there a link? A systematic review and case report analysis. J. Neurol..

[47] Choi H.A., Lee M.J., Choi H., Chung C.S. (2018). Characteristics and demographics of reversible cerebral vasoconstriction syndrome: A large prospective series of Korean patients. Cephalalgia.

[48] Dakay K., McTaggart R.A., Jayaraman M.V., Yaghi S., Wendell L.C. (2018). Reversible cerebral vasoconstriction syndrome presenting as an isolated primary intraventricular hemorrhage. Chin. Neurol. J..

[49] de Boysson H., Parienti J.-J., Mawet J., Arquizan C., Boulouis G., Burcin C., Naggara O., Zuber M., Touzé E., Aouba A. (2018). Primary angiitis of the CNS and reversible cerebral vasoconstriction syndrome: A comparative study. Neurology.

[50] Fugate J.E., Wijdicks E.F., Parisi J.E., Kallmes D.F., Cloft H.J., Flemming K.D., Giraldo E.A., Rabinstein A.A. (2012). Fulminant postpartum cerebral vasoconstriction syndrome. Arch. Neurol..

[51] Li Y., Kaddouh F., Lozano J.D., Jun-O’Connell A., Ramzan M. (2016). Intracerebral Hemorrhage Due to Reversible Cerebral Vasoconstriction Syndrome in the Setting of Antipsychotic Medication (P4. 340). Neurology.

[52] Manning T., Bartow C., Dunlap M., Kiehl R., Kneale H., Walker A. (2021). Reversible Cerebral Vasoconstriction Syndrome Associated With Fluoxetine. J. Acad. Consult. Liaison Psychiatry.

[53] Mikami T., Obata R., Steinberg D.I., Skliut M., Boniece I. (2021). Marijuana-related Reversible Cerebral Vasoconstriction Syndrome. Intern. Med..

[54] Miller T.R., Shivashankar R., Mossa-Basha M., Gandhi D. (2015). Reversible Cerebral Vasoconstriction Syndrome, Part 1: Epidemiology, Pathogenesis, and Clinical Course. Am. J. Neuroradiol..

[55] Noguchi S., Miyaoka R., Miyachi H. (2023). Clinical Images: Symmetrical Gyriform Restricted Diffusion in Severe Reversible Cerebral Vasoconstriction Syndrome. World Neurosurg..

[56] Pham H., Gosselin-Lefebvre S., Pourshahnazari P., Yip S. (2020). Recurrent thunderclap headaches from reversible cerebral vasoconstriction syndrome associated with duloxetine, xylometazoline and rhinitis medicamentosa. CMAJ.

[57] Robert T., Kawkabani Marchini A., Oumarou G., Uske A. (2013). Reversible cerebral vasoconstriction syndrome identification of prognostic factors. Clin. Neurol. Neurosurg..

