# Supplementary material for: Clinical Characteristics of COVID-19-Related Reversible Cerebral Vasoconstriction Syndrome: A Systematic Review of Case Series
Source: J Clin Med. 2025 Jan 14;14(2):487. doi: 10.3390/jcm14020487 (PMC11765861; doi:10.3390/jcm14020487)
Supplement: Supplementary file 1 [file jcm-14-00487-s001.zip › jcm-3380777-supplementary.pdf]

Supplementary Table S1. Search terms for PubMed, SCOPUS and Web of Science

Domain	Search terms
COVID-19	(“COVID-19” OR “SARS CoV-2")
RCVS	AND (“RCVS” OR “Reversible cerebral vasoconstriction syndrome”)

Supplementary Table S2. Cases excluded due to insufficient evidence for RCVS diagnosis

	Age/Sex	Radiologic findings	
		Brain CT/MRI	Brain CTA, MRA, DSA
Ray et al. 2021	64/F	MRI (3 days prior to admission) : few discrete T2/ FLAIR white matter hyperintensities in bilateral centrum semiovale<br><br>MRI (at admission): bilateral cortical and subcortical frontal, parietal, occipital cortical white matter hyperintensities with hemorrhages and diffusion restriction (consistent with PRES)<br><br>MRI (repeated): increase in size of lesions with hemorrhagic transformation and significant edema	MRA (admission): widespread vasoconstriction of intracranial blood vessels suggestive of RCVS<br><br>MRA (repeated): persisting vasospasm in all vessels
Hmaidan et al. 2023	16/F	Head MRI: bilateral parafalcine restricted diffusion<br>spine MRI: unremarkable<br><br>Diffusion-weighted MRI (Day6): bilateral ACA distribution diffusion restriction with new areas of restriction cortically in the insula with concurrent concern for an ACA infarct	N/A
Scheer et al. 2022	56/M	CT: massive, left frontal hemorrhage causing a dramatic midline shift (it was revealed to be massive space-occupying SAH through surgery)	CTA: rarefied vessels on the left side in the area of the ACA, MCA. no vascular malformation and no perfusion alterations were detected.<br><br>postoperative CT: massive stroke in the anterior, middle and posterior cerebral artery supply zone as a result of the postoperative entrapment

Supplementary Table S3. Offending drugs of RCVS

	Number (n = 24)	Percentage (%)
Antidepressants		
Serotonin and Norepinephrine reuptake inhibitors		
Duloxetine	2 / 24	8.3%
Selective serotonin reuptake inhibitors		
Citalopram	3 / 24	12.5%
Escitalopram	1 / 24	4.2%
Fluoxetine	1 / 24	4.2%
Other antidepressants		
Bupropion	1 / 24	4.2%
Mirtazapine	1 / 24	4.2%
Atypical antipsychotics		
Lurasidone	1 / 24	4.2%
Anti-anxiety drug		
Benzodiazepine	1 / 24	4.2%
Anti-epileptic drug		
Barbiturate	1 / 24	4.2%
Carbamazepine	1 / 24	4.2%
Gabapentin	2 / 24	8.3%
Levetiracetam	1 / 24	4.2%
Oxcarbazepine	1 / 24	4.2%
Topiramate	1 / 24	4.2%
Illicit drugs		
Marijuana	3 / 24	12.5%
Opiates	1 / 24	4.2%
Migraine abortive drug		
Acetaminophen with caffeine compound	1 / 24	4.2%
Almotriptan	1 / 24	4.2%
Zolmitriptan	1 / 24	4.2%
Sumatriptan	1 / 24	4.2%
Analgesics		
Ibuprofen	4 / 24	16.7%
Acetaminophen	3 / 24	12.5%
Hormonal agents		
Tamoxifen	1 / 24	4.2%
Progestin contraceptive implant	1 / 24	4.2%
Milrinone	2 / 24	8.3%
Intravenous immunoglobulin	2 / 24	8.3%
Epinephrine	2 / 24	8.3%
Nasal decongestants	1 / 24	4.2%

Antidepressants, antipsychotics, nasal decongestant, migraine remedy, intravenous immunoglobulin, cannabinoid, epinephrine, analgesics, sedatives, epilepsy drugs were considered as offending drugs of reversible cerebral constriction syndrome. packed cells, fluid therapy, corticosteroid (methylprednisolone), denosumab, rosuvastatin, cetirizine were not considered as offending drugs. 17 patients of 24 patients took one or more offending drugs.

Supplementary Table S4. COVID-19 related symptoms

Symptoms	$Number_{case}/Number_{total}$ (total number = 24)	Percentage (%)
Asymptomatic	4 / 24	16.7%
Fever	9 / 24	37.5%
Respiratory Symptoms		
Non-specific respiratory symptoms	2 / 24	8.3%
Cough	7 / 24	29.2%
Dyspnea	5 / 24	20.8%
Hypoxia	4 / 24	16.7%
Rhinorrhea	1 / 24	4.2%
Sore throat	5 / 24	20.8%
Sputum	1 / 24	4.2%
Gastrointestinal Symptoms		
Abdominal pain	1 / 24	4.2%
Diarrhea	3 / 24	12.5%
Dysentery	1 / 24	4.2%
General weakness	5 / 24	20.8%
Poor appetite	1 / 24	4.2%
Malaise	1 / 24	4.2%
Headache	3 / 24	12.5%
Musculoskeletal pain		
Arthralgias	1 / 24	4.2%
Myalgia	1 / 24	4.2%
Anosmia	2 / 24	8.3%
Ageusia	1 / 24	4.2%
Agnosia	1 / 24	4.2%
Diaphoresis	1 / 24	4.2%
Dyspnea on exertion	1 / 24	4.2%
Vertigo	1 / 24	4.2%

Supplementary Table S5. RCVS related Symptoms

Symptoms	$Number_{case}/Number_{total}$ (total number = 24)	Percentage (%)
Headache		
Thunderclap headache	15 / 24	62.5%
Non-specific headache	5 / 24	20.8%
Gastrointestinal symptoms		
Nausea	9 / 24	37.5%
Vomiting	8 / 24	33.3%
Sensory disturbances	5	
Visual impairment	3 / 24	12.5%
Photophobia	3 / 24	12.5%
Phonophobia	1 / 24	4.2%
Hypoesthesia	1 / 24	4.2%
Altered mental status	8	
Confusion	3 / 24	12.5%
Delirium	1 / 24	4.2%
Decreased consciousness	1 / 24	4.2%
Obtundation	2 / 24	8.3%
Unresponsiveness	1 / 24	4.2%
Neurological dysfunctions	4	
Aphasia	2 / 24	8.3%
Ataxia	1 / 24	4.2%
Facial palsy	1 / 24	4.2%
Nystagmus	1 / 24	4.2%
Urinary incontinence	1 / 24	4.2%
Encephalopathy	3 / 24	12.5%
Seizure	1 / 24	4.2%
Paralysis	5	
Hemiplegia	3 / 24	12.5%
Hemiparesis	2 / 24	8.3%
Weakness	2 / 24	8.3%
Lethargy	1 / 24	4.2%
Dysarthria	1 / 24	4.2%

Supplementary Table S6. Involved cerebral vascular segments

	Bilateral	Right only	Left only
Internal carotid artery	2 (1.68%)	0 (0.00%)	2 (1.68%)
Vertebral artery	1 (0.84%)	0 (0.00%)	0 (0.00%)
Vertebrobasilar junction*	1 (0.84%)		
Basilar artery*	3 (2.52%)		
Superior cerebellar artery	1 (0.84%)	0 (0.00%)	0 (0.00%)
Anterior inferior cerebellar artery	2 (1.68%)	0 (0.00%)	0 (0.00%)
Posterior inferior cerebellar artery	1 (0.84%)	0 (0.00%)	1 (0.84%)
Anterior cerebral artery			
A1	3 (2.52%)	0 (0.00%)	0 (0.00%)
A2	8 (6.72%)	2 (1.68%)	0 (0.00%)
A3	0 (0.00%)	0 (0.00%)	1 (0.84%)
Middle cerebral artery			
M1	3 (2.52%)	1 (0.84%)	2 (1.68%)
M2	10 (8.40%)	0 (0.00%)	2 (1.68%)
M3	3 (2.52%)	0 (0.00%)	3 (2.52%)
M4	2 (1.68%)	0 (0.00%)	3 (2.52%)
Posterior cerebral artery			
P1	4 (3.36%)	0 (0.00%)	0 (0.00%)
P2	5 (4.20%)	0 (0.00%)	1 (0.84%)
P3	3 (2.52%)	0 (0.81%)	1 (0.84%)
Diffuse / Multifocal †	1		

Since the total number of cerebral vascular involvement in the radiologic findings of our patient group was 119, the number of cases involving each segment was divided by 119.
Cases with unspecified left/right sides were considered bilateral. Proximal portions were regarded as A1, M1, and P1, and distal portions were regarded as A3, M3, and P3 or more distal.
Anterior circulation vasospasm of #2 patient was considered as involvement of bilateral A1, A2, M1, M2.
Luminal irregularity near the tip of basal artery at the takeoff of the bilateral PCA of #5 patient was considered as involvement of basilar artery and bilateral P1.
Pericallosal artery involvement of #9 patient was considered as A3 involvement.
In patient #10, first CTA showed stenosis in the right P1, but subsequent CTA and DSA showed multifocal narrowing in bilateral PCA, so it was considered that bilateral PCA, not the right P1, was involved.
Involvement of PCA parietal occipital territories of #11 patient was considered as involvement of P3 segment.
Focal narrowing in the left PCA (near the P2-P3 junction) and left MCA (beyond the origin of the superior division) in #14 patient were considered as involvement of left P2, P3, M2.

* Since vertebrobasilar junction and basilar artery are not a blood vessel divided into right and left, they are marked as bilateral
† Since the segment could not be determined with given data, it was written as Multifocal as recorded in the paper.

Supplementary Table S7. Odds ratio analysis for mortality in RCVS.

	OR	95% CI	p-value
Sex, male	N/A	[0.075, N/A]	1
Complication	N/A	[0.160, N/A]	0.482
History of migraine	2.070	[0.024, 178.934]	1
History of hypertension	3.194	[0.036, 281.467]	0.446
Offending drug	N/A	[0.075, N/A]	1

OR = Odds Ratio; CI = Confidence Interval; N/A = Not Available

Supplementary Table S8. Odds ratio analysis for complication in RCVS.

	OR	95% CI	p-value
Sex, male	12.670	[1.119, 712.230]	0.023
History of migraine	1.632	[0.222, 14.282]	0.679
History of hypertension	1.944	[0.212, 26.727]	0.649
Offending drug	1.853	[0.230, 16.982]	0.659

OR = Odds Ratio; CI = Confidence Interval
